# Editorial

**Published:** 1847-01

**Authors:** 


### THE MEDICAL EXAMINER.

PHILADELPHIA, JANUARY, 1847.

### THE BULLETIN OF MEDICAL SCIENCE, EDITED BY JOHN BELL, M. D.

The December number of this Journal contains the *farewell address* of its Editor, in which he announces that "The Select Medical Library and The Bulletin of Medical Science will cease to be published after this date." "These instruments," says the Editor, "through which we have long discoursed with our professional brethren, will no more give utterance to our exhortation, our praises, our monitions and rebukes: our voice, henceforth, comes down to the key and pitch of those who may have, hitherto, listened with some attention, not unmixed, perhaps, with some deference." Those who, like ourselves, have long been in the practice of reading the productions of his ever ready and well pointed pen, will regret that any circumstances should render it necessary for the able and estimable Editor to retire from a position which education and habit so peculiarly qualified him to fill. His personal friends, however, of which number for nearly a quarter of a century we have had the pleasure of considering ourselves one, recollecting his late severe indisposition, and the causes of it, will hope from the change for a longer continuance of his useful labors, than could have been reasonably expected under the excessive mental occupation to which he was exposed. No one probably enjoyed the pleasures that pertain to an Editors position more than Dr. Bell, or better knew its labours and responsibilities, so graphically sketched in the following extract.

"But if, in thus descending from our 'high estate,' we divest ourselves of the appendages of office, and cease to enjoy its honors, its emoluments, and its collateral advantages, we, at the same time, cast off heavy responsibility and ever-recurring anxieties. Those engaged in the busy walks of life have little conception of the toilsome efforts, and the many trying hours encountered by the occupant of the editorial sanctum,—in the task of selection and arrangement of contemporary materials, and of interweaving them with the learning of the past, so as still to preserve a philosophic texture,—a careful analysis and an impartial adjudication of conflicting claims of discovery and improvement,—the support of modest merit,—and the rebuke of arrogant pretensions."

"Ten years have elapsed since the first publication of the Select Medical Library; during which time, it has been the good fortune of its Editor to make it a vehicle for the dissemination, throughout the length and breadth of the land, of works, many of which adorn our medical literature and have contributed, not a little, to the practical instruction of our medical men, at the same time that they have inspired them with more curiosity, and a desire for a still wider circle of reading and research."

The business relations of Dr. Bell and the publishers, Messrs. Barrington and Haswell, are to be continued, and hereafter his labours, instead of being partly occupied with the journal, will be wholly employed on works destined—we hope—to be of a less ephemeral character.

---

### NATIONAL MEDICAL CONVENTION.

In our *Record* department we have given the names of the gentlemen who have been appointed to represent the Medical Faculty of the University of Pennsylvania, the College of Physicians of Philadelphia, and the Philadelphia Medical Society, in the Convention to be held in this city in May next; in all twenty-seven. If all the other medical societies and institutions throughout the country shall be represented in an equal ratio, it will certainly be a large assemblage.

As the period at which the Convention is to take place grows nearer, we discover a more general interest manifested in the subject, and a freer expression of opinion as to the feasibility or otherwise of the various schemes likely to be proposed. On another page we have treated cursorily of some points which it is probable will be mooted, and may perhaps advert to the subject again on some future occasion; in the meanwhile we shall be happy to receive from any of our correspondents, temperate arguments on the questions expected to be raised, and especially those to be reported on by committees appointed at the late Convention, held in the city of New York.

---

### GUN COTTON.

This preparation, it appears, explodes at a temperature as low as 140° of Fahrenheit, which renders it much more dangerous to handle than gunpowder. We have heard of some serious accidents happening to persons of skill and prudence, while engaged with it. In one instance, a letter containing a small portion was blown to atoms, from the heat of the wax used in sealing it. In another case, a chemist, whilst drying a few ounces of it in a current of warm air, not exceeding the temperature of 140 degrees, was badly burnt by its explosion.

This circumstance, together with its comparative costliness, renders it probable that, notwithstanding its tremendous power, it will not supersede the old material for ordinary explosive purposes.

---

### NEW MEDICAL ASSOCIATION.

We are gratified to learn that a number of the physicians residing in the northern section of Philadelphia, embracing Spring Garden, Northern Liberties, Kensington, Germantown, and Frankford, are about forming themselves into an association for literary and scientific improvement, and the promotion of good fellowship and sound medical ethics among the members. It is intended to admit none as members but graduates in medicine, excluding all who are practising or encouraging the various systems of charlatanry, under the mask of reform, and thereby undervaluing the science and skill of regular physicians, and thus bringing the profession into disfavour with the community.

Many of our oldest and most respected physicians reside in the district mentioned, and their association for such praisworthy objects cannot but be productive of good, especially to their junior brethren, who will by this means be admitted to their more intimate fellowship.

---

TO CORRESPONDENTS AND PUBLISHERS.

We have received several books, tracts, and introductory lectures, which we shall endeavour to notice in our next number.

